# Developing and validating a questionnaire for mortality follow-back studies on end-of-life care and decision-making in a resource-poor Caribbean country

**DOI:** 10.1186/s12904-020-00630-0

**Published:** 2020-08-14

**Authors:** Nicholas Jennings, Kenneth Chambaere, Cheryl Cox Macpherson, Karen L. Cox, Luc Deliens, Joachim Cohen

**Affiliations:** 1 End-of-Life Care Research Group, Vrije Universiteit Brussel (VUB), Ghent University, Laarbeeklaan 103, B-1090 Brussels, Belgium; 2 St. George’s University, St. George’s, Grenada; 3 Bioethics Division, St. George’s University School of Medicine and Windward Islands Research and Education Foundation, St. George’s, Grenada; 4 Palliative Care Unit, Caura Hospital, North Central Regional Health Authority, Caura, Trinidad and Tobago; 5 Department of Public Health and Primary Care, Ghent University, Ghent, Belgium

**Keywords:** Questionnaire design, Methodology, Terminal care, Developing country, Caribbean region

## Abstract

**Background:**

Palliative and end-of-life care development is hindered by a lack of information about the circumstances surrounding dying in developing and resource-poor countries. Our aims were to develop and obtain face and content validity for a self-administered questionnaire on end-of-life care provision and medical decision-making for use in population-based surveys.

**Methods:**

Modelled on validated questionnaires from research in developed countries, our questionnaire was adapted to the cultural sensitivity and medico-legal context of Trinidad and Tobago. Two sets of semi-structured face-to-face cognitive interviews were done with a sample of physicians, sampling was purposive. Phase 1 assessed interpretation of the questions, terminology and content of the questionnaire. Phase 2 was tested on a heterogeneous group of physicians to identify and fix problematic questions or recurring issues. Adjustments were made incrementally and re-tested in successive interviews.

**Results:**

Eighteen physicians were interviewed nationwide. Adaptations to questionnaires used in developed countries included: addition of a definition of palliative care, change of sensitive words like expedited to influenced, adjustments to question formulations, follow-up questions and answer options on medications used were added, the sequence, title and layout were changed and instructions for completion were included at the beginning of the questionnaire.

**Conclusion:**

A new instrument for assessing and documenting end-of-life care and circumstances of dying in a small, resource-poor Caribbean country was developed and validated, and can be readily used as a mortality follow-back instrument. Our methods and procedures of development can be applied as a guide for similar studies in other small developing countries.

## Background

“Palliative care is an approach that improves the quality of life of patients and their families facing the problem associated with life-threatening illness” [1]. Substantial progress has been made in the development of palliative and end-of-life care in developed countries, supported by robust population level research including the use of mortality data on the social and clinical circumstances of dying [2]. Such research has led to investment in quality palliative and end-of-life care like choice in the place of care (e.g., private homes, care homes or hospices), quality assurance of end-of-life care in different settings [3, 4], and development of appropriate policies and laws addressing ethical issues around withholding or withdrawing care or even medical aid in dying [5]. The majority of people worldwide lacking effective palliative and end-of-life care live in developing and resource-poor countries [6]. These are countries where the capability to provide care for life-threatening illness is limited due to a lack of necessary human and material resources and consequent services, inadequate education and training for healthcare providers, and a lack of governmental policies to support the provision of palliative care [7, 8]. But these countries are crippled by the slow development of palliative care, reasons for which can include the lack of a research culture and infrastructure, and appropriate instruments and methods to examine palliative care and the circumstances of dying; attitudes of healthcare providers, the person who is dying and those close to them regarding illness, death and dying; opiophobia, which affects the availability and use of opioids for medical use, and suboptimal symptom management [9]. Apart from recent studies on place of death, there is a complete lack of empirical information on the provision of and access to palliative care in Trinidad and Tobago (T&T), including care characteristics at the end of life [10]. The population is aging [11] and is afflicted by a high prevalence of degenerative diseases [12]. Many of those living with conditions like cardiovascular and cerebrovascular diseases and cancer who could potentially benefit from palliative care are likely not to receive it because palliative care is not a public health priority [13]. Knowledge of the nature of care at the end of life including availability, access and quality, and the circumstances influencing medical end-of-life decisions, can provide better insight into the care and treatment people receive before death. An effective way of obtaining this information is by population-wide monitoring using questionnaires supplemented by mortality data [14]. Questionnaires used for population-based surveys on end-of-life care in developed countries cannot simply be applied to developing or resource-poor countries but require adaptation to the context of the culture, ethico-legal circumstances and available healthcare resources of the target country. All questionnaires traced in the literature were developed for developed countries, confirming the need for reliable, culturally sensitive information on end-of-life care in developing and resource-poor countries. To provide a first scientific epidemiological perspective on end-of-life care conditions, the aims of this study were to adapt and obtain face and content validity for a self-administered questionnaire on end-of-life care provision and medical decision-making for use in population-based mortality follow-back surveys in a small Caribbean country like Trinidad and Tobago.

## Methods

### Design

This study concerns the development and cognitive testing of a questionnaire to collect population-based information in a standardised manner on the medical characteristics of palliative care and end-of-life care (i.e., care provided in the last 30-days before death), and the circumstances of dying in a developing country. The resource-poor nature of the setting guided our design rationale. We identified mortality follow-back studies as the preferred method to collect epidemiologic and socio-demographic information at the population level as it permits representative sampling of deaths [15]. We used theoretical guidelines for questionnaire design [16] and empirical pre-testing, specifically cognitive interviewing, a strategy to understand how participants interpret information in relation to their medical practice [17]. The COnsolidated criteria for REporting Qualitative research (COREQ) for cognitive interviewing was used [18]. Relevant issues pertaining to terminal illness and dying in T&T were informed by a systematic review of palliative and end-of-life care in the English-speaking Caribbean [9]. Our questionnaire was modelled on a validated questionnaire on end-of-life care and medical decision-making in the Netherlands [19], variations of which were used in the United Kingdom [20], Belgium [21], New Zealand [22], and Canada [23]. Item generation was done by comparing these four questionnaires. Relevant topics were identified and selected for inclusion in our questionnaire but reformulated to the cultural and medico-legal context of T&T. The principal researcher (NJ), a resident and national of T&T with a public health and clinical nursing background, collated a set of context-relevant closed-ended questions into a draft questionnaire. In consultation with co-authors, (KC, LD and JC) the questions were reviewed and adjustments made prior to seeking comments from the Trinidad and Tobago Medical Association (T&TMA) which reviewed for contextual congruence.

### Setting and sampling

The study was conducted in T&T from November 2017 to March 2018. Since statistical generalization was not intended, sampling was purposive [24], i.e., we deliberately sought participants who were logically assumed to represent the population of physicians with knowledge and experience with end-of-life care. Therefore, a sample of physicians from appropriate medical specialities were recruited by the T&TMA with the inclusion criterion that they provided care to a dying patient within the last year.

### Data collection

To validate the questionnaire, semi-structured face-to-face cognitive interviews were undertaken. Draft questionnaires were tested on the sample of physicians to explore and understand their interpretation of the questions and evaluate the kinds of responses generated by participants [25]. Verbal probing [26] was used to further stimulate conversations on issues regarding a word, phrase or concept. An interview guide, (Online supplemental 1), was specifically designed to systematise the process, debrief and document participant responses after completion. The interview guide and questionnaire were used as triggers to elicit participant discussion which was divided into two parts. Part I contained general questions, not in the questionnaire, intended to capture the overall understanding, of each participant, of the types of questions being asked, and the structure and logical flow of the questionnaire. Part II contained a series of questions relating to the content of the questionnaire and were asked to understand semantic interpretation by participants and the cultural sensitivity of the questions in the T&T context, e.g., were questions clear, difficult to answer, confronting etcetera. Ethics approval to conduct the study was provided by the Ministry of Health of Trinidad and Tobago. Two sets of interviews were done:

### Phase 1

Expert opinion on concepts in the draft questionnaire was sought from specialists experienced in providing palliative care, including general practitioners and hospital-based physicians. Their interpretation of the questions was used to assess terminology and whether the topics were effectively captured. Their comments were integrated into the next iteration of the instrument, which was subsequently reworded and tested with a different group of physicians in phase 2.

### Phase 2

The revised draft questionnaire was tested on a heterogeneous subgroup of physicians with characteristics of interest (sex, region of practice and medical specialty) to identify and fix problematic questions or recurring issues [24]. After completing the questionnaire, any additional relevant feedback on semantics, how the questions related to their practice or what was considered meaningful were obtained, changes were made. Each revised version was used in successive interviews.

### Interview procedure

Prior to all interviews, participants were e-mailed information about the study and its objective i.e., validating the questionnaire. The interview process and duration were also explained, as was the intention to publish findings in a peer-reviewed scientific article, with deletion of any identifiers such as names of places, individuals or facilities. The principal researcher scheduled appointments with participants at their convenience. Participants were individually interviewed in their offices, clinical settings or other mutually agreed locations. At the interview, participants were asked to read and sign a consent form and give their verbal consent to having interviews audio recorded. They were then prompted to think about the last relevant death they had attended. A highlighter pen was offered to mark items on the questionnaire for discussion with the researcher. Time taken to complete the questionnaire and interview were noted.

### Data analysis

Participants were asked to read the questions and relate the scenario to their clinical practice. Information–field notes and audio recordings–from cognitive interviews were systematically documented [24] by the principal researcher. The research team used simple thematic analysis without the use of software to analyse the data. We had group discussions that helped us identify emerging themes, resolve and make systematic decisions on individual problems that arose after interviews. Patterns of problems were grouped, like whether a respondent interpreted a word or question differently than intended, whether an answer option to a question was missing or whether a follow-up question was needed. Incremental adjustments were made after interviews, which were continued until respondents indicated little or no problems with new information, this occurred from the eleventh to eighteenth participant. We made a practical decision that data saturation was reached [27].

## Results

A heterogeneous group of 18 physicians was interviewed nationwide (Table 1), medical specialties of the sample of physicians included, general practitioners, oncologists, gerontologists, internal and palliative medicine. The average interview time was 46 min (range; 17 to 105 min) and the average time to complete the draft questionnaire was eight minutes (range; five to 20 min). One participant opted out of the audio recording.

**Table 1:** Characteristics of participants interviewed

Item	Phase 1 (#)	Phase 2 (#)	Total (#)
**Number of interviews**	5	13	18
**Clinical Specialty**			
General practitioners	3	6	9
Specialists	2	7	9
**Region**			
Trinidad	3	11	14
Tobago	2	2	4
**Sex**			
Male	2	7	9
Female	3	6	9

### General questions

Physicians were asked two broad questions about palliative and end-of-life care to better understand their perceptions of these topics in the cultural context of T&T (online supplemental 2). The first question was ‘what do you think of when I say palliative care?’ Participants responded by mentioning words, phrases or sentences that included goals of care e.g., maintaining comfort or addressing symptom management of terminally ill patients with chronic diseases; improving quality of life by avoiding suffering and assisting the dying through the dying process when a medical cure was not an option; addressing psychosocial issues of the person who was dying and those close to them. The second question was ‘what type of care do you consider as palliative care?’ Some participants saw no difference between this and the previous question. Others framed it as a dimension of care, e.g., physical, spiritual, or emotional, that can include the family. Others identified it as a physical place of care like a hospice. Responses to the relevance of the question and the structure and flow of the questionnaire are summarised in Table 2. Our intention was to follow the order of general questions in our interview guide, but when asked ‘should any of the questions be deleted’ and ‘were any important questions missing’, the order shifted. Participants broadly discussed the meaning of the questions, incorporated their own experiences and remarked on gaps in the healthcare system. This emergent flow was not interrupted but was used as an opportunity to probe participants on these issues.

**Table 2:** Selected participant responses to general questions asked during the validation process (November 2017 – March 2018).

Question	Participant Responses
Is there anything in the questionnaire that does not reflect the clinical realties in a Trinidad and Tobago context?	• Questions appear to reflect American culture and laws. • Questions assume that physicians practice some sort of palliative care. • The questionnaire is generic but suitable for the expertise that exists amongst most physicians. • General practitioners who do not regularly provide EOLC may take a longer time to complete the questionnaire. • Some physicians may not be aware of what the terms withholding and withdrawing treatments are in an EOLC situation. • We do not have a referral pathway for palliative care particularly for physicians not working in institutions. • There is no continuity of care and asking questions that relate to patient care, e.g., ‘within the last 30 days’ becomes difficult to trace or track. • The lack of DNR policies corners physicians to continue doing everything to save life, especially in an institutional setting. • A lack of regulations makes it difficult to practice. • Resources like drugs used in EOLC situations are not radially available, e.g., Propofol, morphine and other opioids, and there is a lack of human resources, e.g., counsellors and home care providers. • There are no on-call palliative care physicians, they are all only by referral. • There are not enough inpatient services to match the number of deaths that may require palliative care. • There are no hospices in Tobago. • There is little formal training for the family to care for patients at home.
Are the questions presented in a logical sequence? If not, how could it be improved?	• Some participants thought the sections on ‘care and treatment’ and ‘medical practice’ should be combined.
Does the questionnaire take too long to complete?	• No, there appears to be a lot of questions but it does not take long to complete.
Are the routing directions (e.g., go to question x) clear enough?	• Yes, instructions are helpful.
Is the layout and organisation confusing? If so, how can it be improved?	• The background colour should be changed. • Font size should be increased.

Abbreviations: EOLC – end-of-life care; DNR – do not resuscitate

### Specific questions

Participant feedback necessitated changes to the content and structure of the draft questionnaire. The validated questionnaire contains twenty-six questions and is organised into five sections (see Appendix 1 for the full questionnaire). The word palliative was removed from the title as some participants were unfamiliar with the term and found it confusing. Several participants had difficulty understanding the terminology of some questions, e.g., some found the filter questions, Q1 (Was this death sudden and totally unexpected?) and Q2 (When was your first contact with the patient?), confusing or irrelevant.

In the section ‘Care and treatment,’ Q3 asked about prioritising treatment goals in the last seven days of life and which was the main goal. Some participants had difficulty defining a main goal but instead identified the need to treat terminal patients symptomatically, e.g., if a patient had an infection, antibiotics would be given. Other participants did not recognise a difference between care and treatment and the subsequent section on medical practice. Although the World Health Organisation has defined palliative care, a more relatable definition was used because many participants had difficulty with questions in which the term was used. The definition from Palliative Care Australia, “Care that helps people live their life as fully and as comfortably as possible when living with a terminal illness. It identifies and treats symptoms which may be physical, emotional, spiritual or social” [28], was chosen because it succinctly described and was aligned to the dimensions of care physicians encountered during their clinical practice.

Under the section ‘Medical practice’ most participants suggested changes were needed to drug categories, e.g., the removal of anaesthetics like Propofol but inclusion of drugs like antiemetics, laxatives, antipsychotics (Chlorpromazine) and benzodiazepines (Diazepam). Most participants also recommended changing the word ‘expedited’ in its relation to the timing of death due to the administration of drugs or withholding or withdrawing of treatment.

The section ‘Decision-making’ required a follow-up question, Q18 (‘why was there no discussion with the patient about the various options related to end-of-life treatments?’) to explain why the patient was not included in the decision-making process.

The final section concerning ‘Information about physicians’ also required a follow-up question regarding competence with communication skills. Q25 (‘do you consider you have enough expertise to communicate adequately with family of patients at the end of life?’). Online supplemental 3 contains a complete list of participant perceptions of questions, comments and changes made to the questionnaire.

## Discussion

### Summary of results

This study demonstrates that despite challenges in doing public health related research in a resource-poor or developing country context, it is possible to do robust studies by using existing instruments developed for developed countries, but it is important to make relevant cultural adaptations. We developed and validated a self-administered questionnaire, to assess and document palliative and end-of-life care and medical decision-making at the end-of-life. Important changes included: the addition of a palliative care definition and instructions for completion; adjustments to question formulations, wording, sequence of questions and answer options and layout; and removal or addition of questions and answer options.

### Strengths and limitations

The questionnaire was validated using face-to-face interviews, which are an effective means of detecting social cues like intonation and body language [29]. To maintain their confidentiality participants were individually interviewed, which enabled unencumbered opinions about issues and questions in the draft questionnaire. Incrementally adjusting the questionnaire when additional relevant feedback was obtained facilitated efficient resolution of important cultural perspectives, problematic words, phrases or issues. By interviewing a heterogeneous mix of physicians whose perspectives represent a range of practices, the questionnaire can be readily used with minimal concern for sensitivity and semantics. Nevertheless, the limited sample typically targeted in cognitively testing questionnaires [26] presents the possibility that some perspectives existing among physicians are not included in this study. Although representativeness for the physician population was not a criterion in this study, but rather variation in their perspectives, it is possible that the physicians interviewed overrepresented those that have some palliative care training or were interested in the topic. Cognitive interviewing is time-consuming for all parties; some interviews were rushed because participants had to return to their clinical duties and information transfer was less effective as a result.

### Implications and interpretation

Our study is the first of its kind in T&T and makes an important contribution to the field by delivering a validated questionnaire for survey studies on end-of-life practices in a resource-poor country. Further reliability and criterion validity studies, e.g., comparison with actual care provision can further research based on the questionnaire. We extracted relevant questions from validated questionnaires used in developed countries, and adapted them to the cultural, social and clinical context of T&T. Our methods and procedures used to validate the questionnaire can be applied in other resource-poor countries when doing similar studies provided there is sufficient local validation. In countries that lack data on the circumstances of death and dying, physicians form a critical data source and population-level research requires their participation [30]. As in a similar study [23], physicians clearly understood the need to modify existing questions relating to end-of-life care practices in developed countries, particularly regarding medication availability and use, to reflect clinical practice, including pain and symptom management. In the absence of any supportive national policy on palliative and end-of-life care, semantic meanings of words like ‘expedited’, or ‘withholding and withdrawing’ life-prolonging treatments, when used in relation to the timing of death, were considered confronting. Physicians interpreted the meaning of such words as an attempt to hasten death. Also evident from interviews was the unfamiliarity of some physicians with the term palliative care, and could be due to a near-total lack of a palliative care culture in medical practice but also to the lack of a substantive palliative care programme at medical schools, the absence of locally available postgraduate training, and the lack of related specialists. This may be detrimental for the quality of medical care delivered to terminally ill patients. To potentially address this issue, physicians can play a key role in advocating for palliative and end-of-life care within their specialty, in lobbying for a palliative care curriculum integrated into existing medical school coursework, and in supporting efforts to improve their palliative care knowledge and skills [31]. When doing health-related research and collecting survey data a number of inherent challenges exist for small developing and resource-poor countries including small populations [32], underdeveloped health infrastructure with a limited range of medical specialists [33], and the lack of a research culture [34]. The latter results in underrepresentation, on the global stage, not only of research from these countries but, importantly, of the epidemiological landscape of their end-of-life care [8].

## Conclusion

This study supports the need to adapt questionnaires used in studies in developed countries to the culture of a developing country. Modelled on existing questionnaires, a new instrument for assessing and documenting end-of-life care in a small, resource-poor, Caribbean country was developed and validated, and is now ready for use. Use of the questionnaire can further end-of-life care practice in T&T by improving information on the circumstances surrounding deaths. Our methods and procedures for developing the questionnaire can be used as a guide for similar validation efforts in other developing Caribbean countries, or in countries outside the Caribbean where palliative and end-of-life care are at similar stages of development, and can enhance the possibility for cross-national comparability of population-level studies. A useful activity in the validation process would be for research teams to discuss diverging or contradictory comments and perspectives and make well-informed decisions as we did with certain sensitive words.

## Supplementary information

**Additional file 1.** Appendix 1. Validated questionnaire

**Additional file 2.** Supplemental 1. Interview guide

**Additional file 3.** Supplemental 2. General questions 1 and 2

**Additional file 4.** Supplemental 3. Cognitive interviews. Participant perception of questions, their suggestions or comments, and changes made to the questionnaire.

## Data Availability

The data of this study are with the first author and are available upon reasonable request.

## Abbreviations

T&T: Trinidad and Tobago
COREQ: COnsolidated criteria for REporting Qualitative research
T&TMA: Trinidad and Tobago Medical Association
DNR: Do not resuscitate
ICU: Intensive Care Unit
