# Supplementary material for: Developing and validating a questionnaire for mortality follow-back studies on end-of-life care and decision-making in a resource-poor Caribbean country
Source: BMC Palliat Care. 2020 Aug 14;19:123. doi: 10.1186/s12904-020-00630-0 (PMC7427774; doi:10.1186/s12904-020-00630-0)
Supplement: Supplementary file 1 — Additional file 1. Appendix 1. Validated questionnaire [file 12904_2020_630_MOESM1_ESM.docx]

**Appendix 1: Validated questionnaire**


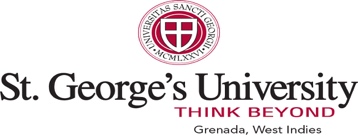


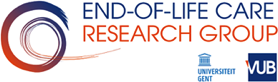


**Confidential Enquiry Into Care And Medical Decision Making At End-Of-Life In Trinidad And Tobago: 2018**

**Dear colleague –** Quality medical care at the end of life is an increasing challenge and there is little to no scientific data on end-of-life care in Trinidad and Tobago. In order to generate objective scientific knowledge, knowledge of your experiences in practice is indispensable.

**Objective –** To **ANONYMOUSLY** survey practicing physicians about their experiences in patient care and medical decision making at the end of life.

**What is asked of you? –** Please try to recall as carefully as possible the patient that is referred to in the accompanying letter, where you were the ***ATTENDING PHYSICIAN*** (if necessary, using the patient’s file or medical records) and complete this questionnaire as accurately as possible. ***Please read the accompanying letter to this questionnaire prior to completing the questionnaire.***

***Research partners:***

- *St George's University School of Medicine, Grenada (SGU)*
- *End-of-Life Care Research Group of the Vrije Universiteit Brussel (VUB) and Ghent University, Belgium.*
- *The Central Statistical Office (CSO) of Trinidad and Tobago*
- *The Trinidad and Tobago Medical Association (T&TMA) and*
- *The North Central Regional Health Authority (NCRHA)*
- *Nicholas Jennings is the primary researcher who is a national and resident of Trinidad and Tobago*

**THERE ARE 26 QUESTIONS IN THIS QUESTIONNAIRE**

**COMPLETING THE QUESTIONNAIRE WILL TAKE YOU ABOUT 2-12 MINUTES**

**PLEASE TICK 🗹 THE BOX OR BOXES TO INDICATE YOUR RESPONSES**

**General**

| **1** | **Was this death sudden and totally unexpected?** | 🞏 Yes: ***go to Question 21 on page 4***  🞏 No |
| --- | --- | --- |
| **2** | **When was your first contact with the patient?** | 🞏 At the time of or after death: ***go to Question 21 on page 4***  🞏 Before death, (please indicate length of time) . . . . . . . . . . . . . . . . . . . . . . . . . |

**Care and Treatment concerning this patient prior to his/her death**

| **3** | **Of the following, which treatment goal was given priority in the last 7 days of life?**  *(Choose only one answer, i.e. the main treatment goal)* | 🞏 Cure  🞏 Prolonging life  🞏 Maintenance of function  🞏 Maximization of comfort  🞏 No overall treatment goals were set  🞏 I have no information regarding the last week of life |
| --- | --- | --- |
| **4** | **Which of the following treatment(s) were given in the last 30 days of life?**  (*multiple answers possible*) | 🞏 Analgesics  🞏 Antibiotics  🞏 Artificial hydration (intravenous line, subcutaneous)  🞏 Artificial nutrition (parenteral nutrition, P.E.G. tube)  🞏 Artificial ventilation (intubation or BPAP)  🞏 Chemotherapy or other cancer therapy  🞏 Cardiopulmonary resuscitation (CPR)  🞏 Dialysis  🞏 Surgery  🞏 Transfusion of blood products  🞏 I have no information regarding the last 30 days of life  🞏 Other, (please specify) . . . . . . . . . . . . . . . . . . . . . . . . . . . . . . . . . . . . . . . . . . . |
| **5** | **In which of the following places did the patient receive care in the last 30 days of life?**  (*multiple answers possible*) | 🞏 At home 🞏 Hospice/palliative care unit  🞏 Ward in hospital 🞏 Geriatric home/Nursing home  🞏 Intensive care unit in the hospital |
| **6** | **Which of the following caregivers, besides yourself, were actively involved in the care for the patient in the last 30 days of life?**  (*multiple answers possible*) | 🞏 Specialist 🞏 Volunteer  🞏 Nurse 🞏 Social worker  🞏 Psychiatrist/psychologist 🞏 Religious/moral counsellor  🞏 Family member 🞏 Religious community member  🞏 Friend 🞏 No one  🞏 I don’t know |
| **7** | **Who, to your knowledge, was present when the patient died?** (*multiple answers possible*) | 🞏 You or another physician 🞏 Friend(s)  🞏 Nurse 🞏 Religious counsellor  🞏 Geriatric home staff 🞏 No one, patient died alone  🞏 Family member(s)  🞏 Other, (please specify) . . . . . . . . . . . . . . . . . . . . . . . . . . . . . . . . . . . . . . . . . . |
| **8** | **What was the patient’s preferred place of death?** | 🞏 At home 🞏 Geriatric home/Nursing home  🞏 Hospital 🞏 I don’t know  🞏 Other, (please specify) . . . . . . . . . . . . . . . . . . . . . . . . . . . . . . . . . . . . . . . . . . |
| **9** | **Did this patient receive any **palliative care* (at home, in a hospital or elsewhere)?**  (*multiple answers possible*) | 🞏 Yes, by a palliative care service 🞏 Yes, by myself and/or other clinician 🞏 Yes, by a family member 🞏 Yes, by a psychologist  🞏 Yes, by a social worker 🞏 No: ***go to Question 11*** |
| **10** | **When did the patient first receive **palliative care* before his/her death?** | 🞏 1-7 days: ***go to Question 12*** 🞏 3-6 months: ***go to Question 12***  🞏 1-4 weeks: ***go to Question 12*** 🞏 More than 6 months: ***go to Question 12***  🞏 1-3 months: ***go to Question 12*** 🞏 I don’t know: ***go to Question 12*** |
| **11** | **For which reason(s) was **palliative care* not initiated?** (*multiple answers possible*)  **** By Palliative Care we mean “Care that helps people live their life as fully and as comfortably as possible when living with a terminal illness. It identifies and treats symptoms which may be physical, emotional, spiritual or social.”*** ^28^ | 🞏 Unaware of how to access palliative care services in my region  🞏 I didn’t think the patient needed palliative care  🞏 A person or service qualified in palliative care, was not available  🞏 There was insufficient time to initiate palliative care  🞏 To not take away hope from the patient or the family  🞏 Financial constraints of the patient or family  🞏 Patient did not want it  🞏 Family did not want it  🞏 Other, (please specify) . . . . . . . . . . . . . . . . . . . . . . . . . . . . . . . . . . . . . . . . . .  . . . . . . . . . . . . . . . . . . . . . . . . . . . . . . . . . . . . . . . . . . . . . . . . . . . . . . . . . . . . . . . .  . . . . . . . . . . . . . . . . . . . . . . . . . . . . . . . . . . . . . . . . . . . . . . . . . . . . . . . . . . . . . . . . |

| **Medical practice** | | |
| --- | --- | --- |
| **12** | **Please indicate whether you prescribed or administered any of the following drugs to treat or alleviate symptoms this patient had in the last 7 days of life?** (*multiple answers possible*) | 🞏 No drugs were used to alleviate symptoms: ***go to Question 14***  🞏 Morphine or other opioid  🞏 Diazepam or other benzodiazepine  🞏 Dopamine or other vasopressors  🞏 Chlorpromazine or other phenothiazines  🞏 Laxative  🞏 Antiemetic  🞏 Other drug (please specify) . . . . . . . . . . . . . . . . . . . . . . . . . . . . . . . . . . . . . . . .  . . . . . . . . . . . . . . . . . . . . . . . . . . . . . . . . . . . . . . . . . . . . . . . . . . . . . . . . . . . . . . . . . |
| **13** | **In your opinion, could the administration of these drugs have influenced the timing of death?** | 🞏 No influence on timing of death: ***go to Question 15***  🞏 Yes, possibly delayed the timing of death: ***go to Question 15***  🞏 Yes, possibly brought forward the timing of death: ***go to Question 15***  🞏 Yes, certainly brought forward the timing of death: ***go to Question 15*** |
| **14** | **Why were no drugs used to alleviate symptoms?**  (*multiple answers possible*) | 🞏 Patient had no significant symptoms  🞏 Drugs were not available  🞏 Financial constraints of the patient or family  🞏 To avoid drug side-effects (life-shortening or addiction)  🞏 Patient refused  🞏 Family refused  🞏 Other, (please specify) . . . . . . . . . . . . . . . . . . . . . . . . . . . . . . . . . . . . . . . . . . . |
| **15** | **Did you withhold and/or withdraw any of the following potentially life-prolonging treatment(s)?** | 🞏 No: ***go to Question 16***  🞏 Yes: if yes, **Please tick the appropriate box(s**) *multiple answers possible* |
| \|  \| Antibiotics \| Artificial hydration (intravenous line, subcutaneous) \| Artificial nutrition (parenteral nutrition, P.E.G. tube) \| Artificial ventilation (intubation or BPAP) \| Chemo-therapy or other cancer therapy \| Dialysis \| Transfusion of blood products \| Cardio-pulmonary resuscitation (CPR) \| Surgery \| \| --- \| --- \| --- \| --- \| --- \| --- \| --- \| --- \| --- \| --- \| \| **Withheld** \| **☐** \| **☐** \| **☐** \| **☐** \| **☐** \| **☐** \| **☐** \| **☐** \| **☐** \| \| **Withdrawn** \| **☐** \| **☐** \| **☐** \| **☐** \| **☐** \| **☐** \| **☐** \|  \|  \| | | |

| **16** | **In your opinion, could the withholding and/or withdrawing of treatments have influenced the timing of death?** | 🞏 No influence on timing of death  🞏 Yes, possibly brought forward the timing of death  🞏 Yes, certainly brought forward the timing of death |
| --- | --- | --- |

**Decision-making**

| **17** | **Did you discuss with the patient the various options related to end-of-life treatments?**  (*multiple answers possible*) | 🞏 Yes, about withholding treatment(s) ***go to Question 19***  🞏 Yes, about withdrawing treatment(s) ***go to Question 19***  🞏 Yes, about giving drug(s) to alleviate pain and/or symptoms ***go to Question 19***  🞏 No, no discussion: ***go to Question 18*** |
| --- | --- | --- |
| **18** | **Why was there no discussion with the patient about the various options related to end-of-life treatments?** (*multiple answers possible*) | 🞏 Patient was unconscious: ***go to Question 19***  🞏 Patient had significant cognitive impairment: ***go to Question 19***  🞏 Patient had a psychiatric disorder: ***go to Question 19***  🞏 Other (please specify) . . . . . . . . . . . . . . . . . . . . . . . . . . . . . . . . . . . . . . . . . . . . |
| **19** | **With whom, of the following, did you discuss the end-of-life treatment options for this patient?** (*multiple answers possible*) | 🞏 With your medical colleague/s  🞏 Nursing staff  🞏 Another caregiver  🞏 The patient’s partner or relatives  🞏 Other person (please specify) . . . . . . . . . . . . . . . . . . . . . . . . . . . . . . . . . . . . . |
| **20** | **As far as you know, did the patient ever express a wish for the end of life to be hastened?** | 🞏 Yes, explicitly  🞏 Yes, but not explicitly  🞏 No  🞏 I don’t know |

**Information regarding yourself as a physician**

| **21** | **What is your gender?** | 🞏 Male 🞏 Female |
| --- | --- | --- |
| **22** | **How long have you practiced as a physician?** | 🞏 under 5 years 🞏 16 to 20 years  🞏 5 to 10 years 🞏 over 20 years  🞏 11 to 15 years |
| **23** | **Have you ever had any formal training in palliative care?** (*multiple answers possible*) | 🞏 No  🞏 Yes, as part of my medical degree  🞏 Yes, postgraduate education programme  🞏 Other training, (please specify) . . . . . . . . . . . . . . . . . . . . . . . . . . . . . . . . . . . . |
| **24** | **Do you consider you have enough expertise to communicate adequately with patients at the end of life?** (*multiple answers possible*) | 🞏 No  🞏 Yes, through experience/informal training  🞏 Yes, through formal training |
| **25** | **Do you consider you have enough expertise to communicate adequately with family of patients at the end of life?** (*multiple answers possible*) | 🞏 No  🞏 Yes, through experience/informal training  🞏 Yes, through formal training |
| **26** | **Do you feel sufficiently supported, by the current protocols of care in Trinidad and Tobago, with your preferred management and decision-making at the end of life for your patient?** | 🞏 No  🞏 Yes  If you wish, please elaborate your response choice here. . . . . . . . . . . . . . . . . . . . . . . . . . . . . . . . . . . . . . . . . . . . . . . . . . . . . . . . . . . . . . . . . . . . . . . . . . . . . . . . . . . . . . . . . . . . . . . . . . . . . . . . . . . . . . . . . . . . . . . . . . . . . . . . . . . . . . . . . . . . . . . . . . . . . . . . . . . . . . . . . . . . . . . . . . . . . . . . . . . . . . . . . . . . . . . . . . . . . . . . . . . . . . . . . . . . . . . . . . . .  . . . . . . . . . . . . . . . . . . . . . . . . . . . . . . . . . . . . . . . . . . . . . . . . . . . . . . . . . . . . . . . . . . . |

| **Thank you for your valued participation. To ensure anonymity of your answers you will need to:**   1. **Place the completed questionnaire in the reply-paid envelope, seal it and post it as soon as possible. (Contact TTPOST; email address:** [***pickup@ttpost.*net**](mailto:pickup@ttpost.net) **or call: 669-5361 EXT 519 or 527 to collect the package) and please provide the following information: Address, Contact Name or Department & Phone Number**  - **It will be received by an independent person in a different location and kept separate.** - **It will not be possible for the researchers or anyone else to use your replies to discover your identity or the identity of the patient on whose care you have reported.** |
| --- |

***It is of course impossible to do justice to all the finer nuances of palliative and end-of-life care in a short questionnaire.***

***If some of your answers require further clarification, please write in the space below.***

. . . . . . . . . . . . . . . . . . . . . . . . . . . . . . . . . . . . . . . . . . . . . . . . . . . . . . . . . . . . . . . . . . . . . . . . . . . . . . . . . . . . . .

. . . . . . . . . . . . . . . . . . . . . . . . . . . . . . . . . . . . . . . . . . . . . . . . . . . . . . . . . . . . . . . . . . . . . . . . . . . . . . . . . . . . . .

. . . . . . . . . . . . . . . . . . . . . . . . . . . . . . . . . . . . . . . . . . . . . . . . . . . . . . . . . . . . . . . . . . . . . . . . . . . . . . . . . . . . . .

. . . . . . . . . . . . . . . . . . . . . . . . . . . . . . . . . . . . . . . . . . . . . . . . . . . . . . . . . . . . . . . . . . . . . . . . . . . . . . . . . . . . . .

. . . . . . . . . . . . . . . . . . . . . . . . . . . . . . . . . . . . . . . . . . . . . . . . . . . . . . . . . . . . . . . . . . . . . . . . . . . . . . . . . . . . . .

. . . . . . . . . . . . . . . . . . . . . . . . . . . . . . . . . . . . . . . . . . . . . . . . . . . . . . . . . . . . . . . . . . . . . . . . . . . . . . . . . . . . . .

. . . . . . . . . . . . . . . . . . . . . . . . . . . . . . . . . . . . . . . . . . . . . . . . . . . . . . . . . . . . . . . . . . . . . . . . . . . . . . . . . . . . . .

. . . . . . . . . . . . . . . . . . . . . . . . . . . . . . . . . . . . . . . . . . . . . . . . . . . . . . . . . . . . . . . . . . . . . . . . . . . . . . . . . . . . . .

. . . . . . . . . . . . . . . . . . . . . . . . . . . . . . . . . . . . . . . . . . . . . . . . . . . . . . . . . . . . . . . . . . . . . . . . . . . . . . . . . . . . . .

. . . . . . . . . . . . . . . . . . . . . . . . . . . . . . . . . . . . . . . . . . . . . . . . . . . . . . . . . . . . . . . . . . . . . . . . . . . . . . . . . . . . . .

. . . . . . . . . . . . . . . . . . . . . . . . . . . . . . . . . . . . . . . . . . . . . . . . . . . . . . . . . . . . . . . . . . . . . . . . . . . . . . . . . . . . . .

. . . . . . . . . . . . . . . . . . . . . . . . . . . . . . . . . . . . . . . . . . . . . . . . . . . . . . . . . . . . . . . . . . . . . . . . . . . . . . . . . . . . . .

. . . . . . . . . . . . . . . . . . . . . . . . . . . . . . . . . . . . . . . . . . . . . . . . . . . . . . . . . . . . . . . . . . . . . . . . . . . . . . . . . . . . . .
