# Supplementary material for: Developing and validating a questionnaire for mortality follow-back studies on end-of-life care and decision-making in a resource-poor Caribbean country
Source: BMC Palliat Care. 2020 Aug 14;19:123. doi: 10.1186/s12904-020-00630-0 (PMC7427774; doi:10.1186/s12904-020-00630-0)
Supplement: Supplementary file 2 — Additional file 2. Supplemental 1. Interview guide [file 12904_2020_630_MOESM2_ESM.docx]

**Supplemental 1. Interview guide**

**
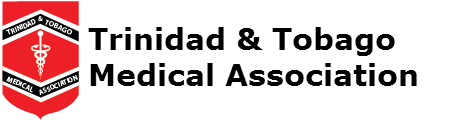

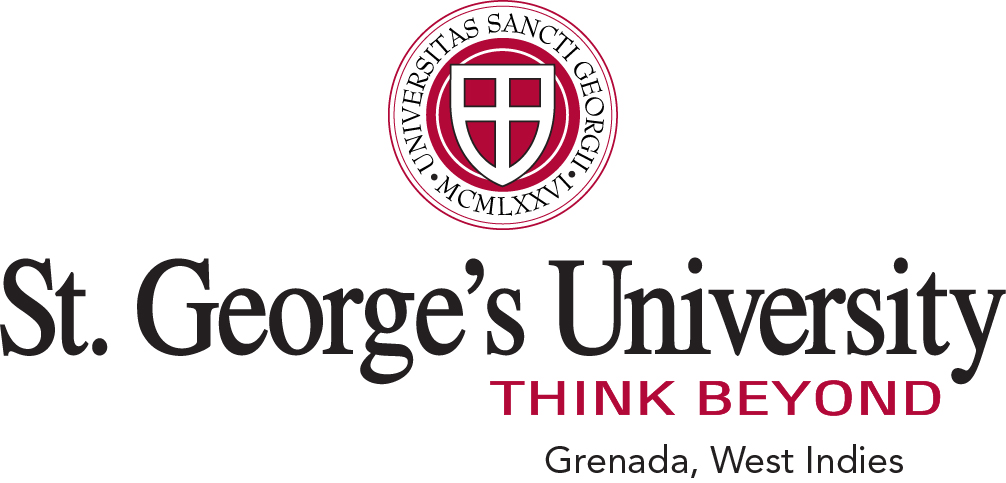

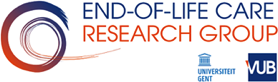
**

**Questionnaire Validation**

**ID number:**

**Date:**

**Questionnaire version: ☐ Phase 1 ☐ Phase 2**

**Participant: ☐ General practitioner ☐ Hospital specialist**

**General remarks:**

**Cognitive testing of an end‐of‐life care and medical end-of-life decision-making questionnaire in a Trinidad and Tobago context**

**INTERVIEW GUIDE**

**(Opening Script)**

You may recall from the Participant Information sheet we sent to you that I would like to ask you to fill out a questionnaire about end-of-life care and medical end of life decision-making in Trinidad and Tobago. The questionnaire is based on a review of existing instruments but has not been validated in the context of Trinidad and Tobago, and we are aiming to do so in this project. We plan on conducting a survey once all the questions are validated. For this project, we are keen to find out how you, as a physician in Trinidad and Tobago, understand and perceive the questions.

There are two steps to this process:

- First, I will like you to complete this questionnaire based on a typical end of life scenario that you might come across in your work (for example, you can think of a specific case you had).
- Second, after you have completed the questionnaire, I will like to ask you about your perceptions of the different questions.

The questions are not designed to elicit personal information about you or circumstances surrounding actual cases you are or have been involved with. Nonetheless, we will treat your responses as confidential. You will be assigned a unique code. Only the research group will have access to your answers, in a way that does not tie your answers back to you, personally, as a respondent. We plan to publish our findings in a peer-reviewed scientific article, but publication of the results will be presented in a way that does not identify any respondent.

If you agree to participate in this interview, and I ask a question that, for whatever reason, you would rather not answer, just say so and we can skip ahead to the next one. Also, if you will like to stop altogether at any point, just say you would like to stop and we can end the interview. If you withdraw partway through, you may ask that your responses be withdrawn from the study.

**Is there any other information you would like to have about this study?**

**No**.

**Yes**: What further information would you like? Answer queries. If you cannot answer queries inform participant you will need to get back to them with the answers. Request time and appropriate phone number to do so.

**Are you willing to participate in this interview now?**

**Yes**: OBTAIN SIGNED CONSENT FORM AND PROCEED.

**No**: DO NOT PROCEED. Thank participant for considering request for interview. Ask if they would be willing to state why they do not wish to participate. Record response. Thank participant for their time and end the interview.

**STEP1:**

**Provide questionnaire to participant**

- Remind them to think about the latest death they attended, (if they are hesitant, ask them to think about an actual case), and to not provide any identifies such as places, or individual or facility names.
- Provide participant with highlighter pen. State: Here is a highlighter pen to assist you to insert a place marker on the questionnaire to remind you about any issues you would like to raise with me after you complete the questionnaire, for example, if you think a question is unclear or does not make sense in a Trinidad and Tobago context.
- Request to record time taken to complete the questionnaire and begin timing.
- Address and record issues if they are raised.

**STEP 2:**

**Conduct post-questionnaire Interview**

I will now like to discuss your perceptions of the questionnaire. The purpose of the questions I am going to ask you is to make sure the survey is capturing the information we want to capture and to get feedback on your experience in completing the questionnaire. I have a list of questions I will like to work though. Some are about the questionnaire in general and some relate to specific questions. Shall I begin asking the questions?

**YES**: Proceed

**NO**: Address any issues they raise. Record issues and your responses. If they do not wish to proceed thank participant for considering request for interview. Thank participant for their time and end the interview.

**PART I – General Questions**

I would like to ask you some general questions that are not part of the questionnaire questions.

1. What do you think of when I say palliative care?

|  |
| --- |

1. What type of care do you consider as palliative care?

|  |
| --- |

1. Is there anything in the questionnaire that does not reflect the clinical realties in a Trinidad and Tobago context?

|  |
| --- |

1. Should any questions be deleted? If so which ones and why?

|  |
| --- |

1. Are any important questions missing? If so, what are they?

|  |
| --- |

1. Are the questions presented in a logical sequence? If not, how could it be improved?

|  |
| --- |

1. Does the questionnaire take too long to complete?

☐ Yes

☐ No

1. Are the routing directions (for example, go to question x) clear enough?

|  |
| --- |

1. Is the layout and organisation confusing? If so, how can it be improved?

|  |
| --- |

**PART II – Specific Interview Questions**

We are interested in your thoughts about the questions posed to you in the questionnaire and will like to spend a little more time on them.

| **No.** | **Evaluation** | **Comments** (Own comments in brackets) |
| --- | --- | --- |
| 1 | - Clear - Confusing / unclear - Difficult to answer - Unimportant - Confronting - Important answer options missing |  |
| 2 | - Clear - Confusing / unclear - Difficult to answer - Unimportant - Confronting - Important answer options missing |  |
| 3 | - Clear - Confusing / unclear - Difficult to answer - Unimportant - Confronting - Important answer options missing |  |
| 4 | - Clear - Confusing / unclear - Difficult to answer - Unimportant - Confronting - Important answer options missing |  |
| 5 | - Clear - Confusing / unclear - Difficult to answer - Unimportant - Confronting - Important answer options missing |  |
| 6 | - Clear - Confusing / unclear - Difficult to answer - Unimportant - Confronting - Important answer options missing |  |
| 7 | - Clear - Confusing / unclear - Difficult to answer - Unimportant - Confronting - Important answer options missing |  |
| 8 | - Clear - Confusing / unclear - Difficult to answer - Unimportant - Confronting - Important answer options missing |  |
| 9 | - Clear - Confusing / unclear - Difficult to answer - Unimportant - Confronting - Important answer options missing |  |
| 10 | - Clear - Confusing / unclear - Difficult to answer - Unimportant - Confronting - Important answer options missing |  |
| 11 | - Clear - Confusing / unclear - Difficult to answer - Unimportant - Confronting - Important answer options missing |  |
| 12 | - Clear - Confusing / unclear - Difficult to answer - Unimportant - Confronting - Important answer options missing |  |
| 13 | - Clear - Confusing / unclear - Difficult to answer - Unimportant - Confronting - Important answer options missing |  |
| 14 | - Clear - Confusing / unclear - Difficult to answer - Unimportant - Confronting - Important answer options missing |  |
| 15 | - Clear - Confusing / unclear - Difficult to answer - Unimportant - Confronting - Important answer options missing |  |
| 16 | - Clear - Confusing / unclear - Difficult to answer - Unimportant - Confronting - Important answer options missing |  |
| 17 | - Clear - Confusing / unclear - Difficult to answer - Unimportant - Confronting - Important answer options missing |  |
| 18 | - Clear - Confusing / unclear - Difficult to answer - Unimportant - Confronting - Important answer options missing |  |
| 19 | - Clear - Confusing / unclear - Difficult to answer - Unimportant - Confronting - Important answer options missing |  |
| 20 | - Clear - Unimportant - Confronting - Important answer options missing |  |
| 21 | - Clear - Confusing / unclear - Difficult to answer - Unimportant - Confronting - Important answer options missing |  |
| 22 | - Clear - Confusing / unclear - Difficult to answer - Unimportant - Confronting - Important answer options missing |  |
| 23 | - Clear - Confusing / unclear - Difficult to answer - Unimportant - Confronting - Important answer options missing |  |
| 24 | - Clear - Confusing / unclear - Difficult to answer - Unimportant - Confronting - Important answer options missing |  |
| 25 | - Clear - Confusing / unclear - Difficult to answer - Unimportant - Confronting - Important answer options missing |  |
| 26 | - Clear - Confusing / unclear - Difficult to answer - Unimportant - Confronting - Important answer options missing |  |
