# Supplementary material for: Developing and validating a questionnaire for mortality follow-back studies on end-of-life care and decision-making in a resource-poor Caribbean country
Source: BMC Palliat Care. 2020 Aug 14;19:123. doi: 10.1186/s12904-020-00630-0 (PMC7427774; doi:10.1186/s12904-020-00630-0)
Supplement: Supplementary file 3 — Additional file 3. Supplemental 2. General questions 1 and 2 [file 12904_2020_630_MOESM3_ESM.docx]

**Supplemental 2. General Questions: Numbers 1 and 2**

**
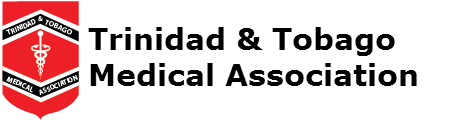

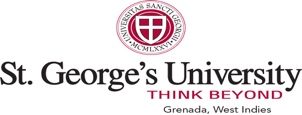

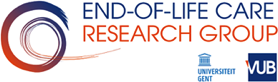
**

| **Question 1: What do you think of when I say palliative care?** | **Question 2: What type of care do you consider as palliative care?** |
| --- | --- |
| “Care when cure is no longer possible.” | “Everything from social, spiritual, financial, emotional, physical, family support . . .” |
| “ . . . providing care to patients with serious illness . . . at any point of the disease . . . to relieve symptoms . . . the patient may not have long to live.” | “All the care provided to relieve symptoms . . . including physical and mental stress, for the patient and family.” |
| “Care of patients with advanced and incurable illnesses . . . any disease that can shorten a patient’s life . . . “ | “Both comfort and supportive care.” |
| “End-of-life care . . . doesn’t necessarily mean withholding treatment . . . but looking at a terminal disease and symptom management related to advanced disease.” | “ . . . could be at different levels . . . for example at a hospice, it could be managed by a palliative care physician . . . it could also be care that is managed by a family physician, or oncologist . . . involves managing symptoms related to disease.” |
| “Preparing for death and to meet your maker. . . there is no cure . . . comfort, pain relief, support, dealing with the personal issues in terms of death and dying.” | Caring for persons with “any terminal illness like AIDS, diabetes, autoimmune diseases.” |
| “The preparation of human mind and body as it comes towards the end of life. . . palliative care starts differently for different patients. . . sometimes while someone is undergoing. . . active management they may need psychological palliative care support prior to switching over from active management into palliative management medically. . . other patients may need active and palliative management at the same time”. | “. . . If we are not actively treating a patient to remission and or cure, then the rest is termed palliation . . . if we are not seeking a cure we have to psychologically prepare a patient for the possibility of end of life. . . certainly symptom control and the medical management surrounding that”. |
| “End-of-life care . . . to alleviate suffering and pain, symptomatic relief more that curative”. | “To provide the basic humanitarian aid like analgesia, food and hydration”. |
| “Palliative care is doing whatever is necessary to improve the quality of life in patients with a terminal illness where cure is not a viable option”. | “Looking at patients with terminal illness, like patients with cancer and non-cancer conditions that medical therapy has little role for in terms of prolonging life (patients with heart failure, renal impairment, muscular paralysis”. |
| The patient is in the best comfort at the end stage of life when we cannot treat the underlying cause of their illness. Focus on supportive care and comfort. | Care when the underlying pathology is untreatable. |
| “Working with patients who are terminally ill, and trying to get people to accept that this is something we cannot cure . . . I use the phrase, landing the plane as gently as possible. By people I mean patients and their relatives, patients take a while to imagine that the horizon is a lot closer than they imagine”. | “I define it by aim of treatment not by interventions. . . for example, chemotherapy in stage 4 cancer is effectively a palliative exercise. . . some people associate palliative care only with the use of opioids but I don’t. I think opioids can be used anytime through the spectrum of illness, similarly, chemotherapy has its place but not right at the end”. |
| “Comfort care and quality of life”. | “Holistic care, not only for the patient’s physical symptoms but also their emotional and psychological symptoms as well and also care for the family”. |
| “Care of anybody with a condition that can’t be cured . . . I’m concerned with people’s quality of life and happiness . . . not focusing only on the death and dying aspect . . . the goal is to help people die well, a good death”. | “Palliative care should start as you get a bad diagnosis from your GP or a specialist physician for a life limiting condition . . . people think it’s really medical but it involves discussions like telling people what to expect for the future, that the disease course is uncertain especially with the none cancer diseases to really mentally prepare people . . . yes there is symptom control but the real hard stuff is the discussions from the time somebody has a bad diagnosis. |
| “Care for the dying. By definition is supportive care, non-curable . . . what we refer to in general practice as trying to avoid suffering, to minimise pain and suffering while the condition or disease process takes its natural course for patients who are beyond any cure or treatment”. | “Pain relief, adequate nutrition, psychological support, management of antecedent things like the chronic complications of bedrest, for example, bedsores”. |
| “A clinical framework which attempts to discern the end of life preferences of the client, in terms of identifying a policy or a living will as to how they will like to prepare for the terminal phase for a chronic disease, and not necessarily confined to cancer. Especially in the knowledge that at some point of a chronic disease, as clinicians, we do not necessarily improve the outcome but we prolong the life, we prolong the life without improving health, with the knowledge that there is a certain timespan to the terminal phase of any disease process and since we can reasonably determine when the terminal phase will be, it’s in my opinion seems reasonable to plan with the patient on how to prepare for the terminal phase ”. | “Palliative care attends to the physical needs, the psychological needs, it also focuses on the caregiver and support of the caregiver, as what I refer to the spiritual or existential need of the patient. It’s trying to balance all these things for good quality of life or reasonable quality of life at the end. Of course, it requires a lot of things, an assessment of the patient, it requires communication, it requires an advanced plan, a management plan of how you are going to do it, which should be dynamic because things may change, the expectations of the client and family may change, there is also the issue of active verses passive actions, doing active things to change an outcome such as instituting care at the end and knowing what the patient wants, e.g.; to be intubated, to receive chest compressions, to receive medications and their effects. Also, who has the last say, your proxy, a living will or both”. |
| “The patient is at end stage disease which will contribute to the patient’s death in a relatively short space of time”. | “Speaking from the medical and psychological point of view, palliative care includes pain control, hydration, looking after the organs in the best way possible, trying to control the blood pressure (comorbidities e.g.; diabetes), maintaining a quality of life for the patient until death. Also, to ensure psychologically the patient, family and caregivers to go through the process of accepting death”. |
| “Support, people dying with dignity, care for terminal patients at the end of life, inclusive of caregivers (family, formal and informal), bereavement and financial planning”. | “Care at the end of life, palliative, hospice, supportive care for any kind of illness that is incurable, e.g.; in oncology, symptom management like nausea and vomiting, making referrals and appointments, helping patient cope”. |
| “Keeping the patient comfortable in the later stages of life usually when there is no curative mechanism available for her condition”. | “Includes an entire spectrum including social, emotional, psychological, spiritual, pain relief, everything we can do to make them comfortable. Curative care is outside the palliative care spectrum”. |
| “Making life as comfortable for someone who has an illness that will limit his/her own life. Comfort care when cure is not possible. Holistic care that involves families as well as patients”. | The participant thought that this question was a repeat of the pervious one and may not yield any additional information. |
