# Supplementary material for: Developing and validating a questionnaire for mortality follow-back studies on end-of-life care and decision-making in a resource-poor Caribbean country
Source: BMC Palliat Care. 2020 Aug 14;19:123. doi: 10.1186/s12904-020-00630-0 (PMC7427774; doi:10.1186/s12904-020-00630-0)
Supplement: Supplementary file 4 — Additional file 4. Supplemental 3. Cognitive interviews. Participant perception of questions, their suggestions or comments, and changes made to the questionnaire. [file 12904_2020_630_MOESM4_ESM.docx]

**Supplemental 3: Cognitive interviews. Participant perception of questions, their suggestions or comments, and changes made to the questionnaire.**

| **Section** | **Question #** | **Draft questions** | **Issue/participant suggestion or comment** | **Change or justification for no change** |
| --- | --- | --- | --- | --- |
| General | 1 | Was this death sudden and totally unexpected? | - Confusing question because the patient is terminal and receiving palliative care, and asking if the death was sudden and unexpected is contradictory, consider keeping ‘sudden’ only. - Consider reversing the order of questions 1 and 2. - The question is confusing, consider replacing it with ‘Were you the attending physician prior to the death of this individual’. | - No change. Question intended to identify persons dying suddenly and less likely to have received end-of-life care. - No change – filter question from patient to physician. - Issue was addressed by including in the cover page instructions stating that the questionnaire is intended for the ‘attending physician’. |
|  | 2 | When was your first contact with the patient? | - Answer option is asking two responses ‘before or at the time of death’. - Include space for physicians to write the time they had known the patient. | - Changed response option to ‘at the time of or after death’. - Change made. |
| Care and Treatment concerning this patient prior to his/her death | 3 | Of the following, which treatment goal was given priority in the last 7 days of life?  *(Choose only one answer, i.e. the main treatment goal)* | - Participants thought it difficult to select only one answer option to this question. - Consider including an answer option for the promotion of relative/s of caregiver wellbeing. | - No change made. Our aim was to get the primary treatment goal for the patient and having multiple answer options would have defeated this purpose. |
|  | 4 | Which of the following treatment(s) were given in the last 30 days of life?  (*multiple answers possible*) | - Answer options are standards of care in an ICU setting. Add analgesics to the answer options as the previous question mentioned maximization of comfort as an answer option. | - Included analgesics as the first main answer option. |
|  | 5 | In which of the following places did the patient receive care in the last 30 days of life?  (*multiple answers possible*) | - Consider adding an answer option ‘I don’t know’. | - No change made. An I don’t know option was deferred to avoid this being a default option. |
|  | 6 | Which of the following caregivers, besides yourself, were actively involved in the care for the patient in the last 30 days of life?  (*multiple answers possible*) | - Remove answer option ‘volunteer’ since there are not many volunteers. Social worker is more applicable to the hospital setting. | - No change made. Answer options can be used in future national surveys and comparisons can be made cross nationally. |
|  | 7 | Who, to your knowledge, was present when the patient died?  (*multiple answers possible*) | - Older persons requiring palliative care are often in geriatric homes, consider adding a response option to include ‘geriatric home staff’. - Consider adding a space for who the family member is/was in the answer options. | - Added answer option ‘geriatric home staff’. - No change made. Answer option ‘other’ can serve this purpose. |
|  | 8 | What was the patient’s preferred place of death? | - Reposition this question as it flows better from question #7. | - Change made. |
|  | 9 | Did this patient receive any *palliative care* (at home, in a hospital or elsewhere)?  (*multiple answers possible*) | - Include a definition of palliative care in the questionnaire. - A participant found it odd to see a footnote (the definition of palliative care) in a questionnaire and did not read it. | - *Added a definition of palliative care (questions 9 through 11 specifically mention palliative care). - The definition’s font colour was made different from the rest of the text. |
|  | 10 | When did the patient first receive palliative care before his/her death? | - No issues with this question | - No change required. |
|  | 11 | For which reason(s) was *palliative care* not initiated? (multiple answers possible) | - Answer option ‘palliative care was not or insufficiently meaningful’ is confusing, consider removing and replacing it with ‘unaware palliative care services exist’ or ‘unaware how to access palliative care services in my region’. - Consider adding ‘Financial constraints’ to answer options. - Answer option ‘palliative care was not or insufficiently meaningful’ was unclear. Consider including directions at the beginning of the questionnaire to remind physicians that responses are from their experience and knowledge of what occurred. | - Changed response option to ‘unaware how to access palliative care services in my region’. - ‘Financial constraints’ added to answer options. - This answer option was removed from the questionnaire. Text was added at the beginning of the questionnaire to address this concern: ‘What is asked of you? – Please try to recall as carefully as possible this death, where you were the ATTENDING PHYSICIAN (if necessary, using the patient’s file or medical records) and complete this questionnaire as accurately as possible. Please read the accompanying letter to this questionnaire prior to completing the questionnaire’. |
| Medical Practice | 12 | Please indicate whether you prescribed or administered any of the following drugs to treat or alleviate symptoms this patient had in the last 7 days of life? (multiple answers possible) | - Consider adding drug options such as; laxatives, antiemetic and removing Propofol and lidocaine; include answer option for a drug category for dementia or restless patients e.g.; chlorpromazine; include an answer option for Diazepam. - Leave more space for writing ‘other’ drugs used. | - These changes were made to the questionnaire. - Additional space added. |
|  | 13 | In your opinion, could the administration of these drugs have influenced the timing of death? | - The word “expedited” is confronting and is understood as hastening death, rephrase to ‘transitioning the death’. | - Recurring issue, the word was changed from ‘expedited’ to ‘influenced’. |
|  | 14 | Why were no drugs used to alleviate symptoms?  (multiple answers possible) | - Consider adding ‘Financial constraints’ to answer options. | - ‘Financial constraints’ added to answer options. |
|  | 15 | Did you withhold and/or withdraw any of the following potentially life-prolonging treatment(s)?  (multiple answers possible) | - None of the response choices apply to home care. - Consider including a timeframe for this question e.g., 14 days. - There should be a follow up question to #15 to ask why did the physician withhold treatment, with answer options 1) No benefit to the patient 2) Financial constraints of the patient or family 3) To save the patient discomfort from invasive treatment. | - No changes made. Suggestions are beyond the scope of this study. - No changes made. Suggestions are beyond the scope of this study. |
|  | 16 | In your opinion, could the withholding and/or withdrawing of treatments have influenced the timing of death? | - The use of ‘expedited’ in the question is a word not often used in the local context, consider changing. - Response option “yes, certainly expedited the timing of death” likely not to be selected by physicians. | - Changed the word ‘expedited’ to ‘influenced’. - The word ‘expedited’ was removed from answer options and replaced with ‘brought forward’. |
| Decision-Making | 17 | Did you discuss with the patient the various options related to end-of-life treatments?  (multiple answers possible) | - A follow-up question to #17 as to why there was no discussion with the patient is missing. | - Question 18 was added. |
|  | 18 | Why was there no discussion with the patient about the various options related to end-of-life treatments? (multiple answers possible) | - Consider adding an answer option, ‘relatives did not want the physician to discuss with the patient’. - Include more space to write for answer option ‘other’. | - No change made as there is an answer option for ‘other’. - Additional space included for ‘other’. |
|  | 19 | With whom, of the following, did you discuss the end-of-life treatment options for this patient? (multiple answers possible) | - In the answer options, consider splitting “The patient’s partner or relatives” into two separate options. | - No change made. Answer option ‘other’ can serve this purpose. |
|  | 20 | As far as you know, did the patient ever express a wish for the end of life to be hastened? | - No issues with this question. | - No change required. |
| Information regarding yourself as a physician | 21 | What is your gender? | - No issues with this question. | - No change required. |
|  | 22 | How long have you practiced as a physician? | - The question ‘how long have you practiced as a specialist in patient care’ is confusing, consider changing to specialist in your field for both hospital physicians and general practitioners. | - The question was rephrased to ‘how long have you practiced as a physician’. |
|  | 23 | Have you ever had any formal training in palliative care? (multiple answers possible) | - Consider removing ‘palliative care for physicians’ from 3rd answer option. - Consider asking physicians whether they want training in palliative care. | - Change made. - No change. Beyond the scope of this study. - Researchers added the option for multiple answers to the question. |
|  | 24 | Do you consider you have enough expertise to communicate adequately with patients at the end of life? (multiple answers possible) | - Answer option ‘no’ is confronting. - Include a response to indicate having ‘some experience’ or ‘some training’. | - No change to answer options made but changed the ordering of responses by moving ‘no’ from last to first option:   - No   - Yes, through experience/informal training   - Yes, through formal training - Question was rephrased from ‘*Do you consider you have enough expertise to deal with patients at the end of life’*. - Researchers added the option for multiple answers to the question. |
|  | 25 | Do you consider you have enough expertise to communicate adequately with family of patients at the end of life?  (multiple answers possible) |  | - Question added – researchers added a follow-up question to #24. |
|  | 26 | Do you feel sufficiently supported, by the current protocols of care in Trinidad and Tobago, with your preferred management and decision-making at the end of life for your patient? | - This question isn’t applicable as there are no laws regarding end-of-life decision making in Trinidad and Tobago. - In the question, consider changing ‘healthcare regulations’ to ‘protocols of care’ | - Question was rephrased from *‘Did your perception of the law, as it applies in Trinidad and Tobago, inhabit or interfere with your preferred management and decision-making at the end of life for your patient’*. - Change made to question. |

**Abbreviations: ICU – Intensive Care Unit;**

***By Palliative Care we mean “Care that helps people live their life as fully and as comfortably as possible when living with a terminal illness. It identifies and treats symptoms which may be physical, emotional, spiritual or social.”^28^**
